# In Vitro Insights into the Role of 7,8-Epoxy-11-Sinulariolide Acetate Isolated from Soft Coral *Sinularia siaesensis* in the Potential Attenuation of Inflammation and Osteoclastogenesis

**DOI:** 10.3390/md22020095

**Published:** 2024-02-19

**Authors:** Lin-Mao Ke, Dan-Dan Yu, Ming-Zhi Su, Liao Cui, Yue-Wei Guo

**Affiliations:** 1 Guangdong Provincial Key Laboratory of Research and Development of Natural Drugs, School of Pharmacy, Guangdong Medical University, Zhanjiang 524023, China; klm102198@163.com (L.-M.K.); ddyu@baridd.ac.cn (D.-D.Y.); 2 Shandong Laboratory of Yantai Drug Discovery, Bohai Rim Advanced Research Institute for Drug Discovery, Yantai 264117, China; 3 School of Medicine, Shanghai University, Shanghai 200444, China

**Keywords:** 7,8-epoxy-11-sinulariolide acetate, RAW264.7 cells, inflammation, osteoclastogenesis

## Abstract

The balance between bone-resorbing osteoclasts and bone-forming osteoblasts is essential for the process of bone remodeling. Excessive osteoclast differentiation plays a pivotal role in the pathogenesis of bone diseases such as rheumatoid arthritis and osteoporosis. In the present study, we examined whether 7,8-epoxy-11-sinulariolide acetate (Esa), a marine natural product present in soft coral *Sinularia siaesensis*, attenuates inflammation and osteoclastogenesis in vitro. The results indicated that Esa significantly inhibited lipopolysaccharide (LPS)-induced inflammation model of RAW264.7 cells and suppressed receptor activator for nuclear factor-κB ligand (RANKL)-triggered osteoclastogenesis. Esa significantly down-regulated the protein expression of iNOS, COX-2, and TNF-α by inhibiting the NF-κB/MAPK/PI3K pathways and reducing the release of reactive oxygen species (ROS) in RAW264.7 macrophages. Besides, Esa treatment significantly inhibited osteoclast differentiation and suppressed the expression of osteoclast-specific markers such as NFATC1, MMP-9, and CTSK proteins. These findings suggest that Esa may be a potential agent for the maintenance of bone homeostasis associated with inflammation.

## 1. Introduction

Bone homeostasis is maintained by a balance between bone-resorbing osteoclasts and bone-forming osteoblasts. Osteoclasts are responsible for matrix degradation and deterioration in bone biology and are well-known as the principal functional cells in bone resorption [1]. Excessive bone resorption will lead to bone loss in skeletal diseases such as osteoporosis, osteolytic bone metastasis, and rheumatoid arthritis. Generally, these bone diseases are mainly associated with abnormal activation of osteoclasts. It has been reported that osteoclast activity is precisely regulated by the cytokine receptor activator for nuclear factor-κB ligand (RANKL) [2]. Many cytokines, for example, interleukin (IL)-1β, IL-6, TNF-α, and so on, increase the RANKL secretion that finally binds to the receptor activator for nuclear factor-κB (RANK) protein on the osteoclast precursors and promotes osteoclastogenesis [3]. Inflammation is also a very important part that is related to the bone disease process. The macrophage–osteoclast axis plays an important role in the interaction between immune cells and bone cells [4]. Pro-inflammatory cytokines secreted by macrophages may regulate the expression of RANKL in the bone microenvironment [5]. As a result, the nuclear factor kappa B (NF-κB) and related signaling pathways are activated and regulate the maturation of osteoclasts, which ultimately leads to the disruption of bone homeostasis in the body and then causes bone disease [6]. Inhibiting osteoclastogenesis or decreasing the bone resorption activity of mature osteoclasts is beneficial to the alleviation of bone resorption-related diseases [7].

Conservative drug therapies such as bisphosphonates, calcitonin, estrogen, and nonsteroidal anti-inflammatory drugs are widely used to treat osteoporosis and related fractures [8]. However, it has been reported that these pharmacological therapies have an inhibitory effect on the synthesis of the bone matrix, and the long-term use of these drugs for treatment increases the risk of hepatorenal toxicity or gastrointestinal intolerance [9,10,11,12,13]. Thus, it is urgent to develop new drug candidates with improved therapeutic potential and better safety profiles for the treatment of osteoporosis.

Marine natural products are universally acknowledged as the metabolites characterized by unique chemical structure and diverse biological activities and attract increasing attention in the study of degenerative inflammatory diseases [14,15]. In the course of our ongoing program toward the search for novel and bioactive metabolites from marine organisms, we have reported a series of new sesterterpenes, diterpenoids, alkaloids, and so on [16,17]. The compound 7,8-epoxy-11-sinulariolide acetate (Esa) is a diterpenoid isolated from the soft coral *sinularia siaesensis* by our group, and it was first isolated from the formosan soft coral *Sinularia flexibilis* by Hsieh in 2003 [18]. It attracted our attention when it revealed a significant anti-inflammation potential. Accumulating evidence indicates that molecules participate in immune regulation, which is highly involved in bone homeostasis [19]. As a potent anti-inflammatory agent with beneficial effects, it is of great interest to us whether Esa can eventually affect the differentiation process of osteoclasts through immune regulation. In this study, we intentionally focused on the biological effect of Esa in LPS-induced inflammation model of RAW264.7 cells and its mechanisms impacting osteoclast differentiation.

## 2. Results

### 2.1. Determination of Cytotoxicity of Esa

**Figure 1:**
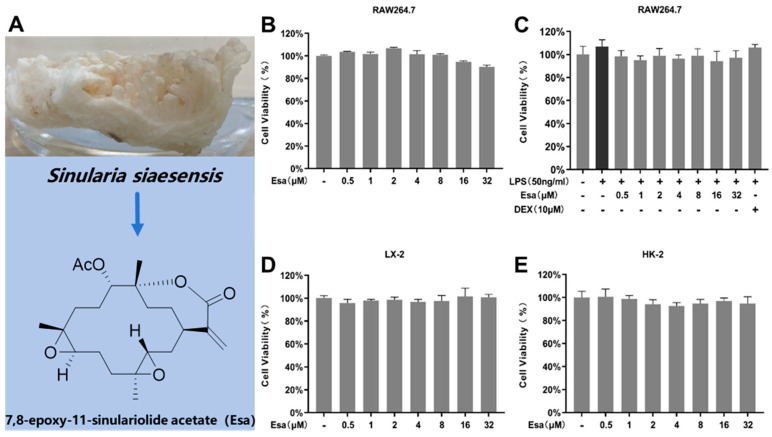
Cell viabilities of RAW264.7, LX-2, and HK-2 cells treated with Esa. (**A**) *Sinularia siaesensis* and the structure of Esa. (**B**) RAW264.7 cells were treated with Esa for 24 h at concentrations from 0.5 μM to 32 μM. (**C**) RAW264.7 cells were pre-treated with Esa at concentrations from 0.5 μM to 32 μM for 1 h and co-cultured with LPS 50 ng/mL for 24 h. (**D**) LX-2 cells were treated with Esa for 24 h at concentrations from 0.5 μM to 32 μM. (**E**) HK-2 cells were treated with Esa for 24 h at concentrations from 0.5 μM to 32 μM. Cell proliferation ratios are the means ± SDs (*n* = 3).

Esa is a marine natural product (Figure 1A). The ^1^H NMR, ^13^C NMR, and MS data of Esa can be found at Supplementary Materials. The potential cytotoxicity of Esa to murine RAW264.7 macrophages, human hepatocyte LX-2, and human renal cell HK-2 was assessed by 3-(4,5-Dimethylthiazol-2-yl)-2,5-diphenyltetrazolium bromide (MTT)assay. The results showed that Esa had no obvious inhibitory effect on these cell viability at the concentrations from 0.5 μM to 32 μM after a 24 h drug treatment (Figure 1B,D,E). To evaluate the cell viability of RAW264.7 cells, after Esa and LPS co-treatment for 24 h, the cells were pre-treated with various concentrations of Esa or dexamethasone (DEX) for 1 h and then were added to LPS solution to make a final concentration of 50 ng/mL. The results revealed that LPS treatment could slightly promote the proliferation of RAW264.7 cells. The cell viability had no significant changes during the Esa and LPS co-treatment for 24 h. These results were compared with that of DEX at a concentration of 10 μM that served as a positive control in the anti-inflammatory activity assay (Figure 1C). No significant change in cell viability was induced by Esa up to 32 μM, which excluded the possibility that reduced cell viability was responsible for the observed decrease in pro-inflammatory proteins. Furthermore, the low cytotoxicity of Esa toward hepatocyte LX-2 and renal HK-2 cells demonstrated its potential safety, an important factor for a lead development compound.

### 2.2. Esa Inhibited the Messenger Ribonucleic Acid (mRNA) and Expression of Pro-Inflammatory Proteins in RAW264.7 Cells

**Figure 2:**
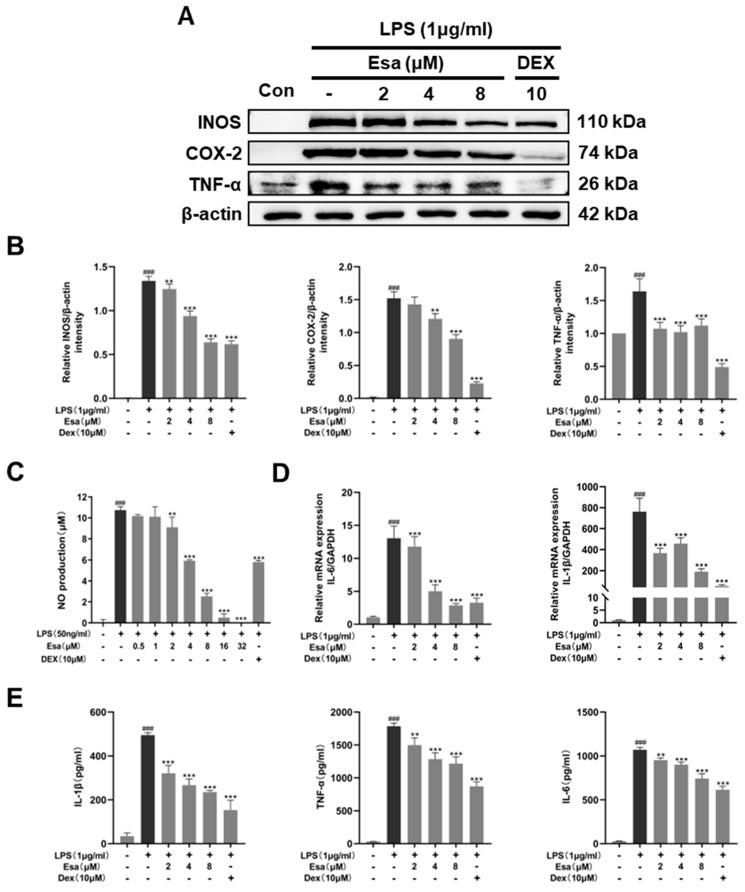
Effect of Esa on LPS-induced inflammation in vitro. (**A**,**B**) RAW264.7 cell were pre-treated with the indicated concentrations of Esa or dexamethasone (DEX) for 1 h, and then with LPS (1 μg/mL, 24 h). The iNOS, COX-2, and TNF-α protein levels were determined by Western blot. (**C**) RAW264.7 macrophages were pre-treated with Esa or dexamethasone (DEX) for 1 h and then with LPS (50 ng/mL, 24 h), and NO concentrations in medium were determined using the Griess method. (**D**) RAW264.7 cells were pre-treated with the indicated concentrations of Esa or dexamethasone (DEX) for 1 h, and then treated with LPS (1 μg/mL, 1 h). Gene expression of IL-6 and IL-1β was measured by qRT-PCR. (**E**) RAW264.7 cells were pre-treated with the indicated concentrations of Esa or dexamethasone (DEX) for 1 h, and then treated with LPS (1 μg/mL, 24 h), before IL-1β, IL-6, and TNF-α production in the cell culture supernatant was analyzed using ELISA kits. The results shown are representative of three independent experiments. ^###^ *p* < 0.001 vs. untreated controls. ** *p* < 0.01, and *** *p* < 0.001 vs. LPS-treated cells.

In order to elucidate the role of Esa in inflammation, the protein levels of pro-inflammatory factors, including inducible nitric oxide synthase (iNOS), cyclooxygenase 2 (COX-2) and tumor necrosis factor (TNF-α), in RAW264.7 cells were analyzed by Western blot. As shown in Figure 2A,B, the levels of these proteins in the LPS-challenged group were significantly increased compared to those in control group, while Esa significantly inhibited the expression of these inflammatory proteins. Since nitric oxide (NO)is a downstream mediator of iNOS, the NO levels in RAW264.7 cell supernatants were measured using Griess reagent. As expected, the NO levels were significantly increased by LPS treatment, but these increases were inhibited by Esa pre-treatment in a dose-dependent manner (Figure 2C). Besides, the transcription levels of pro-inflammatory factors such as interleukin-6 (IL-6) and cytokine interleukin-1 (IL-1β) were also examined by qRT-PCR. It was found (Figure 2D) that the mRNA levels of these pro-inflammatory mediators were increased obviously when RAW264.7 macrophages were exposed to LPS for 1 h, and that these increases were significantly diminished by pre-treatment with Esa in a concentration-dependent manner. Notably, at a concentration of 8 µM, Esa significantly inhibited the mRNA levels of IL-1β and IL-6 to extents comparable to those of 10 µM dexamethasone. Meanwhile, the IL-1β, IL-6, and TNF-α production in the cell culture supernatant was also analyzed using ELISA kits, as shown in Figure 2E; Esa significantly inhibited the promotion of these cytokines stimulated by LPS treatment.

### 2.3. Esa Reduced Reactive Oxygen Species Levels in RAW264.7 Cells

**Figure 3:**
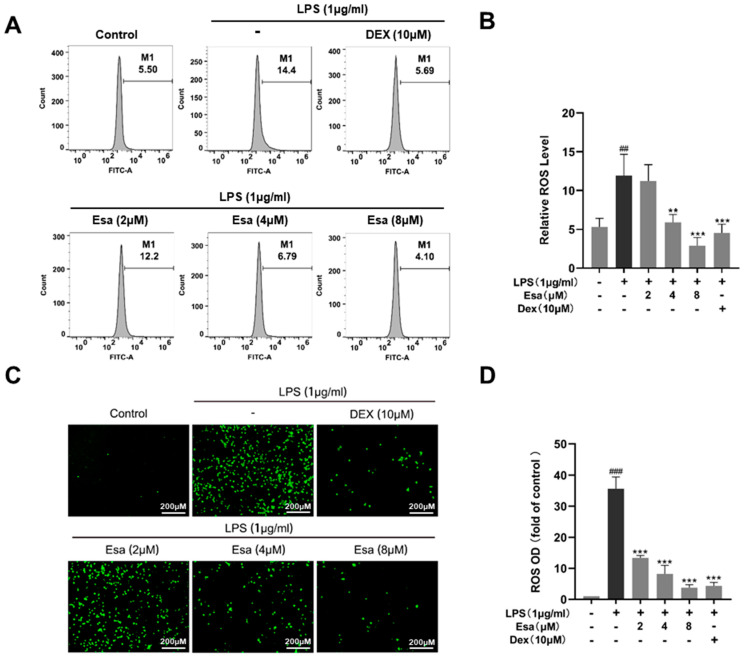
Effect of Esa on ROS level of RAW264.7 cells. (**A**,**B**) ROS levels in RAW264.7 cells were detected and quantified using flow cytometry. (**C**,**D**) ROS levels in RAW264.7 cells were observed by a fluorescence microscope. LPS (1 μg/mL) induced intracellular ROS, which was shown as green fluorescence using the fluorescent probe 2’,7’-Dichlorodihydrofluorescein diacetate (DCFH-DA). ^##^ *p* < 0.01, and ^###^ *p* < 0.001 vs. untreated controls. ** *p* < 0.01, and *** *p* < 0.001 vs. LPS-treated cells.

Reactive oxygen species (ROS) are chemically reactive molecules containing oxygen. In a biological system, ROS are formed as a natural product of the normal metabolism of oxygen. High ROS content will cause oxidative damage to cells and plays an important role in inflammatory responses. Therefore, we detected and quantified cellular oxidative stress using a fluorescence labeling technique and flow cytometry, respectively. In Figure 3A,B, the results of the flow cytometry demonstrated that ROS-positive cells significantly increased in the LPS-treated group compared with the control group, but Esa reduced the ROS positive-cell numbers in a dose-dependent manner. As shown in Figure 3C,D, Esa significantly and dose-dependently reduced ROS production, compared with the LPS treatment group. Notably, at a concentration of 4 μM, Esa significantly decreased ROS production, with a potency comparable with that of 10 μM DEX.

### 2.4. Lipopolysaccharide (LPS)-Induced Nuclear Factor Kappa B (NF-κB) Signaling Was Repressed by Treatment with Esa

**Figure 4:**
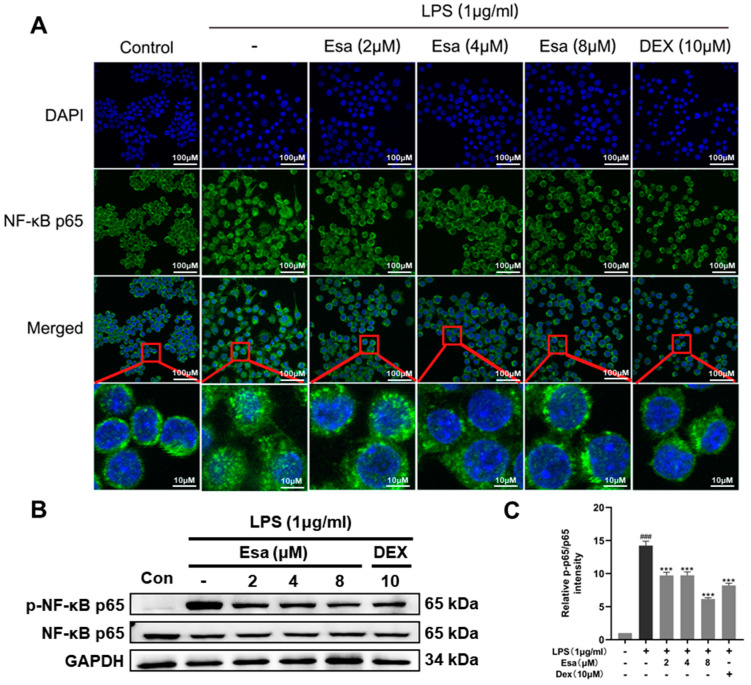
Effect of Esa on NF-κB activation in RAW264.7 cells. (**A**) NF-κB p65 was visualized using confocal microscopy as green fluorescence and the cell nucleus was viewed as blue fluorescence by DAPI staining. The area where the red square is magnified. (**B**,**C**) Phosphorylation of NF-κB. The cells were pretreated with Esa for 1 h and then stimulated with lipopolysaccharide (LPS, 1 μg/mL) for 24 h. The results shown are representative of three independent experiments. ^###^ *p* < 0.001 vs. untreated controls. *** *p* < 0.001 vs. LPS-treated cells.

It is well known that LPS induces ROS production and then influences transcription through the regulation of the phosphorylation of transcription factors. Among the transcription factors, nuclear factor-kappa B (NF-κB) is a critical promoter involved in inflammation and in the induction of pro-inflammatory proteins such as iNOS, COX-2, IL-6, TNF-α, and so on. Since Esa significantly attenuated the ROS production in LPS-induced RAW264.7 cells, we speculated that Esa inhibited NF-κB activity through the reduction of ROS and the subsequent blocking of the NF-κB transcriptional activity of the pro-inflammatory mediators. To investigate the mechanism that is responsible for the effect of Esa on LPS-induced NF-κB signaling, the location of NF-κB p65 in RAW264.7 macrophages were examined under laser confocal microscopy. P65 is a key subunit of NF-κB and its nuclear translocation represents the activating NF-κB. As shown in Figure 4A, the NF-κB p65 subunit was significantly translocated into the cell nucleus after LPS (1 μg/mL) treatment for 15 min, indicating that LPS exposure promotes activation of the NF-κB that will participate in the transcription process of pro-inflammatory molecules. With a treatment of 2–8 μM Esa, p65 entry from the cytoplasm into the nucleus was markedly inhibited, implying that Esa could significantly blunt these changes, especially in the 4 μM and 8 μM groups. The protein levels of the NF-κB p65 and phosphorylated p65 subunits were further evaluated using Western blot; the protein levels of NF-κB p65 did not change obviously in each group, but the protein levels of phosphorylated p65 showed a remarked increase when treated with LPS, indicating the activation of NF-κB pathway. Esa pre-treatment attenuated the activation of NF-κB signaling in a dose-dependent way (Figure 4B,C).

### 2.5. Mitogen-Activated Protein Kinase (MAPK) and Phosphoinositide 3-Kinases (PI3K) Signaling Pathways of Lipopolysaccharide (LPS)-Induced Activation Were Suppressed by Treatment with Esa

**Figure 5:**
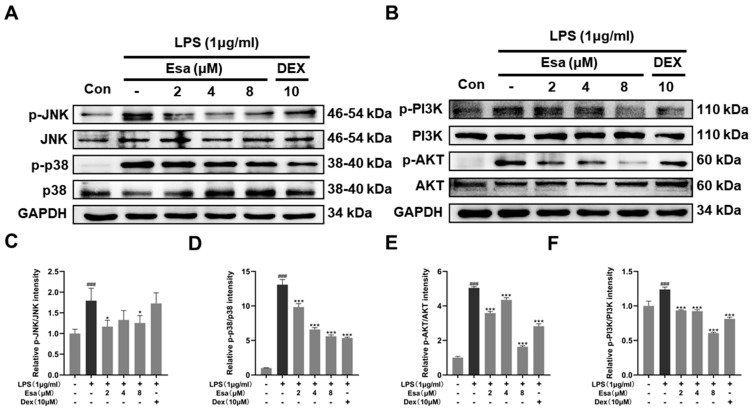
Effects of Esa on MAPK and PI3K signaling pathways in LPS-stimulated RAW264.7 macrophages. RAW264.7 cells were pretreated with Esa or DEX with the indicated concentration for 1 h and then co-cultured with LPS (1 μg/mL) for 24 h. (**A**–**C**) The protein levels and phosphorylation levels of JNK and p38. (**D**–**F**) Protein levels and phosphorylation levels of PI3K and AKT. The results shown are representative of three independent experiments. ^###^ *p* < 0.001 vs. untreated controls. * *p* < 0.05, *** *p* < 0.001 vs. LPS-treated cells.

It has been reported that the PI3K/Akt and MAPK signaling pathways were other pathways upstream of the NF-κB pathway and were related to NF-κB activation when inflammation occurs. In order to verify whether the MAPK and PI3K/Akt signaling pathways were involved in the process of Esa-mediated alleviation of inflammation, RAW264.7 cells were pretreated with Esa for 1 h and then challenged with LPS for 24 h. The protein levels of c-jun N-terminal kinase (JNK), phosphorylated c-jun N-terminal kinase (p-JNK), p38 mitogen-activated protein kinase (p38), phosphorylated-p38 mitogen-activated protein kinase (p-p38), phosphoinositide 3-kinases (PI3K), phosphorylated-phosphoinositide 3-kinases (p-PI3K), protein kinase B (AKT), and phosphorylated-protein kinase B (p-AKT)in the cells were measured. As shown in Figure 5A–C, the JNK and p38 phosphorylation levels increased when the cells were stimulated by LPS without any protections. The treatment of Esa prominently reduced the phosphorylation of JNK and p38. Additionally, the PI3K and its downstream target AKT were fully phosphorylated by the LPS stimulation, while the Esa treatment produced an obvious inhibition in their phosphorylation level at the same time (Figure 5D,E).

### 2.6. Esa inhibited Osteoclastogenesis

**Figure 6:**
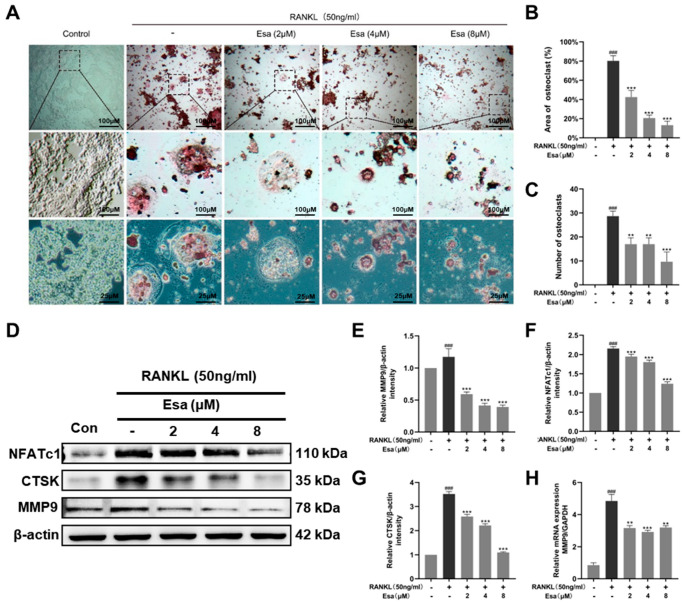
Esa suppressed osteoclast differentiation in vitro. (**A**) Representative images of TRAP staining showing that Esa inhibited osteoclastogenesis dose-dependently in RAW 264.7 cells. The area where the black square is magnified. (**B**,**C**) Quantification of the TRAP-positive multinucleated cells and area of osteoclast (%). (**D**–**G**) Representative Western blot images of the effects of Esa on NFATc1, CTSK, and MMP9 expressions. (**H**) Quantification of mRNA level of MMP9. The results shown are representative of three independent experiments. ^###^ *p* < 0.001 vs. untreated controls. ** *p* < 0.01, *** *p* < 0.001 vs. LPS-treated cells.

Generally, tartrate-resistant acid phosphatase (TRAP) is recognized as a primary marker of osteoclastogenesis as it is highly expressed in differentiated osteoclasts. On the one hand, the RANKL-induced osteoclast differentiation of RAW264.7 macrophages model was used to assess the effect of Esa on TARP activity. As shown in Figure 6A–C, the treatment with RANKL 50 ng/mL on RAW264.7 cells increased the number of TRAP-positive and multi-nucleated cells, compared with that of untreated control cells. Esa treatment decreased the number of TRAP-positive multi-nucleated cells in a dose-dependent manner under these conditions. On the other hand, the effect of Esa and RANKL on the expression levels of the proteins that participated in osteoclast differentiation in RAW264.7 cells was also evaluated. The treatment with RANKL 50 ng/mL induced the expression of NFATc1, CTSK, and MMP9 in RAW264.7 cells. However, Esa significantly down-regulated the expression levels in a dose-dependent manner (Figure 6D–G). In addition, Esa could inverse the effect on mRNA levels of MMP-9 treated with RANKL in a dose-dependent manner in RAW264.7 cells (Figure 6H).

### 2.7. Esa Inhibited Receptor Activator for Nuclear Factor Kappa B Ligand (RANKL)-Induced Mitogen-Activated Protein Kinase (MAPK) and Nuclear Factor Kappa B (NF-κB) Signaling Cascades in RAW264.7 Cells and Promoted Peroxisome Proliferator-Activated Receptor Gamma (PPAR-γ) Protein Levels

**Figure 7:**
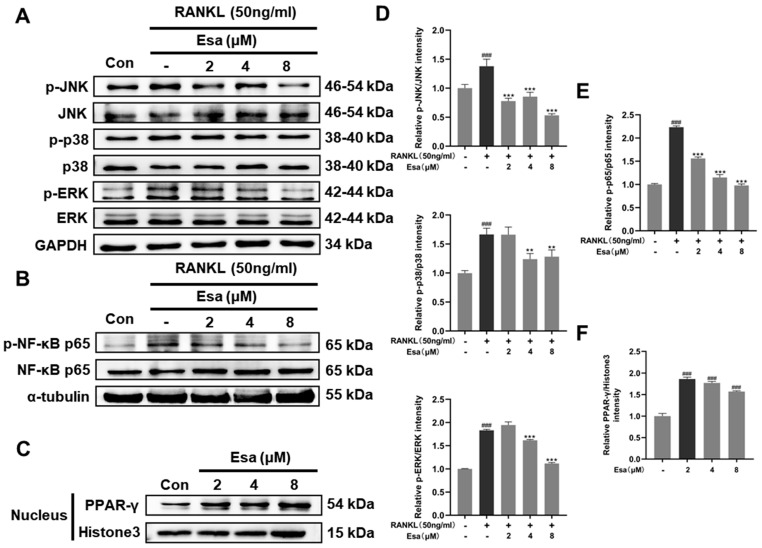
Effect of Esa treatment on signaling pathways during osteoclastogenesis. (**A**,**D**) Esa inhibited the activation of MAPK pathway in RANKL-stimulated conditions. (**B**,**E**) Esa inhibited the activation of NF-κB pathway in RANKL-stimulated conditions. (**C**,**F**) Esa induced PPAR-γ over-expression in RAW264.7 cell nuclei. ^###^ *p* < 0.01 vs. untreated controls. ** *p* < 0.01, *** *p* < 0.001 vs. RANKL-treated cells.

To better understand the mechanisms by which Esa inhibits osteoclast differentiation, Western blot analysis was performed to measure the expression of key factors involved in the osteoclast differentiation process in RAW264.7 cells. As shown in Figure 7A,D, the treatment of Esa significantly suppressed the phosphorylation of JNK, p38, and ERK, which are the representative molecules involved in the MAPK signaling pathway. As expected, the treatment with Esa significantly inhibited the activation of NF-κB pathway by suppressing the RANKL-induced phosphorylation of NF-κB p65 in RAW264.7 cells (Figure 7B,E).

Peroxisome proliferator-activated receptor-γ (PPAR-γ) is a pivotal contributor to immunoregulation. Previous evidence suggests that the NF-κB dependent pathway is related to PPAR-γ activation [20]. In this study, we determined the influence of Esa on PPAR-γ expression in RAW264.7 cells. As expected, this small molecule up-regulated the PPAR-γ expression in the cell nucleus in a dose-dependent manner (Figure 7C,F). These results indicated that Esa inhibits osteoclastogenesis by suppressing MAPK and NF-κB signaling, in relation to PPAR-γ activation, to some extent.

## 3. Discussion

Many inflammatory diseases stem from macrophage activation. Generally, infection, tissue damage, or exposure to endotoxins could cause activated M1 macrophages following the production of various pro-inflammatory mediators, such as inducible nitric oxide synthase iNOS, NO, COX-2, TNF-α, IL-1β, and IL-6. Accumulating evidence indicates that inflammation regulates bone resorption and osteoclastogenesis [21,22,23]. Macrophages are also highly involved in osteoclast-related pathogenesis because they are the responsible cells for the secretion of pro-inflammatory proteins in bone biology [24]. It was reported that pro-inflammatory cytokines, such as TNF-α, IL-1β, and IL-6, may regulate the expression of RANKL, which directly contributed to the differentiation of osteoclasts. Moreover, TNF-α was reported to increase the number of osteoclast precursors or active RANKL, and also to promote bone loss and osteoclastogenesis. Accordingly, these studies implied that osteoclastogenesis-induced bone loss may be inhibited by suppressing inflammation. Our study showed that Esa suppressed the activation of M1 macrophages in RAW264.7 cells. M1 macrophages are well known as a promoter responsible for the release of pro-inflammatory cytokines and exert a negative influence in many bone diseases. Therefore, this study demonstrated that the suppressive effect of Esa on inflammation and subsequent osteoclast activation was caused by preventing macrophage polarization.

Generally, NF-κB works as a main balancer between osteoclast-induced bone loss and osteoblast-induced bone formation. The up-regulation of NF-κB signaling will promote the secretion of pro-inflammatory cytokines such as TNF-α, IL-1β, IL-6, and so on. Subsequently, those pro-inflammatory cytokines further stimulate the NF-κB pathway, as well as osteoclast precursors, intensifying the inflammatory process and osteoclastogenesis. The down-regulation of osteoclast-related genes of the NF-κB pathway, including NFATc1 and CTSK, is an important mediator of the differentiation of the maturation and functions of osteoclasts.

MAPK and PI3K are two other important signaling pathways that participate in the inflammation process [25,26]. They play a critical role in facilitating NF-κB activation [27]. Therefore, compounds that can inhibit these two pathways may have great potential in suppressing the progression of inflammatory reaction, osteoclastogenesis, and diseases related to bone loss. The results in this study revealed that pre-treatment with Esa inhibited the phosphorylation of JNK and p38, which are the key proteins in the MAPK signaling pathway, as well as PI3K and AKT, proteins belonging to the PI3K signaling pathway. In short, the attenuation of inflammatory reactions induced by pre-treatment with Esa is possibly associated with the inhibition of the MAPK and PI3K signaling cascades.

The receptor RANK belongs to the tumor necrosis factor receptor family and regulates bone resorption and bone formation [28,29]. Here, we used its ligand RANKL to induce the differentiation of RAW264.7 into osteoclasts and evaluated the effect of Esa on TRAP activity and osteoclast differentiation [30]. It is easy to see that Esa (2~8 μM) significantly suppressed TRAP activity and osteoclast differentiation. Furthermore, once RANKL binds to the RANK protein, its downstream signaling, especially the NF-κB and MAPK signaling pathways, is activated [31,32,33,34]. Then, activated NF-κB and p38 induce the overexpression of NFATc1, which is a key factor in regulating osteoclastogenesis. Subsequently, other osteoclast-related factors, such as CTSK and MMP-9, are also up-regulated. Hence, we examined these protein levels in relation to osteoclast differentiation via the Western blot method. As expected, Esa significantly inhibited the RANKL-induced up-regulation of NFATc1, CTSK, and MMP-9. Furthermore, the mechanisms underlying the suppressive effects of Esa on osteoclast differentiation were also investigated. When RANKL induced the activation of the NF-κB and MAPK signaling pathways, Esa pre-treatment inhibited them significantly. Thus, these results demonstrate that Esa is an effective inhibitor of osteoclastogenesis, due to its suppressive effects on the expression of NFATc1 and the activation of the NF-κB and MAPK pathways.

Recently, the PPAR-γ protein has attracted considerable attention as a therapeutic target in the treatment of various diseases. It is a ligand-activated transcription factor belonging to the nuclear hormone receptor superfamily, and it is commonly known that PPAR-γ plays a crucial role in the regulation of glucose homeostasis, adipocyte proliferation, cell cycle control, carcinogenesis, and atherosclerosis. Accumulating evidence indicates that the activation of PPAR-γ can down-regulate inflammation as well as osteoclastogenesis. It was reported that PPAR-γ agonists, including troglitazone, pioglitazone, and rosiglitazone, showed inhibitory effects on osteoclast differentiation and bone resorption activity in vitro [35,36,37,38]. Hassumi et al. showed that the well-known PPAR-γ agonist rosiglitazone may be able to reduce alveolar bone loss in ligature-induced periodontitis in a rat model, by decreasing RANKL expression and inhibiting osteoclast differentiation [39]. It is a particular focus for scientists to establish a strategy to prevent bone destruction triggered by inflammation, as in periodontal disease and rheumatoid arthritis. Our results indicated that Esa could promote the expression of PPAR-γ in a dose-dependent manner. The increased expression of PPAR-γ has a close relationship with the inhibition of the NF-κB-mediated inflammatory reaction and the suppression of RANKL-induced osteoclast differentiation, to some extent. Taking these into account, we speculated that Esa could serve as a potential therapeutic agent that inhibits RANKL-induced MAPK and NF-κB signaling cascades possibly related to PPAR-γ.

RANKL-induced RAW264.7 macrophages are widely used as in vitro models of osteoclast differentiation [40,41], and Esa indeed exhibited significant attenuation effects on osteoclast differentiation in this model. However, murine primary bone marrow macrophages and human CD14-positive cells are ideally also used as models for in vitro evaluation, and further studies are necessary to clarify the in-depth mechanism of Esa regarding bone destruction occurring in inflammatory bone loss lesions in animal models. As a marine natural product, only 5 mg of Esa was obtained by the isolation and purification of soft corals in our laboratory. Due to the small amount of compound produced, this study, which was required to examine Esa’s therapeutic potential in osteoporosis disease, were limited. Nonetheless, to our knowledge, this is the first study to clarify the molecular regulatory mechanisms of Esa in osteoclast differentiation. The chemical synthesis of Esa and its derivatives would be very valuable in the development of marine-derived anti-osteoporosis drugs and is one of our ongoing works.

## 4. Materials and Methods

### 4.1. Chemicals and Reagents

The natural product Esa was isolated from the soft coral *Sinularia siaesensis*. Briefly, the dry animals (427 g) were cut into small pieces and were extracted exhaustively with acetone at room temperature (4 × 2.0 L). The acetone extract was partitioned into aqueous methanol (MeOH) and diethyl ether. The diethyl ether portion was concentrated and followed with silica gel column chromatography separation, as well as reverse phase HPLC purification. Finally, a pure compound (5 mg) was identified as 7,8-epoxy-11-sinulariolide acetate by comparison with proton and carbon nuclear magnetic resonance (^1^H-NMR,400 MHz; ^13^C-NMR, 125 MHz), and ESI-MS data with the literature.

The following antibodies were used: COX-2, iNOS, TNF-α (from Affinity, Jiangsu, China), p-PI3K, PI3K, p-AKT, AKT, p-p65, p65, ERK, p-ERK, p-p38, p38, p-JNK, JNK, MMP9 (from Cell Signaling Technology, Danvers, MA, USA), NFATc1, CTSK, PPAR-γ (from Santa Cruz Biotechnology, Dallas, TX, USA), and GAPDH and β-actin (from proteintech, Shanghai, China). Anti-rabbit horseradish-linked IgG was used as the secondary antibody. Signals were developed using the ChemiDoc™ Touch Imaging System (Bio-Rad Laboratories, Hercules, CA, USA). Dulbecco’s Modified Eagle Medium (DMEM) and fetal bovine serum (FBS) were purchased from Gibco (from Thermo Fisher Scientific, Waltham, MA, USA). Penicillin–streptomycin–gentamicin solution (from Solarbio Science & Technology Co., Ltd., Beijing, China), MTT reagent (from Adamas-beta, Shanghai, China), and LPS (from Sigma-Aldrich, Darmstadt, Germany) are commercially available reagents. 

### 4.2. Cell Culture

The murine macrophage cell line RAW264.7, human hepatic astrocyte LX-2, and human renal cortical proximal convoluted tubular epithelial cell line HK-2 were obtained from the National Collection of Authenticated Cell Cultures of China. Cells were maintained in DMEM high glucose medium supplemented with 10% FBS and antibiotics (100 mg/mL streptomycin, 2.5 mg/L amphotericin B) and cultured at 37 °C in a 5% CO_2_ humidified incubator.

### 4.3. 3-(4,5-dimethylthiazol-2-yl)-2,5-diphenyl-2H-tetrazolium bromide (MTT) Assay

RAW264.7 cells, LX-2 cells, and HK-2 cells were seeded in 96-well plates at a density of 5 × 10^3^ cells per well and cultured overnight, and then treated with different concentrations of Esa for 24 h. The cell viabilities were evaluated using MTT reagent, which was added to each well (5 mg/mL, 20 μL) and incubated at 37 °C for 4 h avoiding the light, and then the supernatant was aspirated, and 150 μL dimethyl sulfoxide (DMSO)was added to each well. Absorbances were measured using a microplate reader at a wavelength of 570 nm.

### 4.4. Production Level of Nitric Oxide (NO) in Cell Supernatants

RAW264.7 cells were seeded in a 96-well plate at a density of 3 × 10^4^ cells per well for 12 h. Cells were pre-treated with various concentrations of Esa or dexamethasone for 1 h and then co-incubated with 50 ng/mL of LPS from *Escherichia coli* O111:B4 for 24 h. NO concentrations in medium were determined using a Griess assay. Griess reagent (70 µL) was added to media supernatants (70 µL) and then incubated at 37 °C for 15 min avoiding the light. Absorbance was measured at 520 nm. NO concentrations were calculated using 0–100 µM sodium nitrite standards.

### 4.5. Quantitative Real-Time Polymerase Chain Reaction

RAW264.7 cells were seeded in 6-well plates at a density of 2 × 10^5^ cells per well and cultured overnight. Cells were pre-treated with different concentrations of Esa for 1 h, co-incubated with 1 µg/mL of LPS for 1 h, and then total RNA was isolated using Trizol reagent (Invitrogen, Carlsbad, CA, USA), and first strand cDNA was synthesized using total RNA and the Fast All-in One-RT Kit (Es Science, Shanghai, China). cDNA products and primers for each gene were used for PCR with AccuPower^®^ PCR PreMix (BIONEER, Seoul, Republic of Korea) in a BIO-RAD T100™ Thermal Cycler PCR unit (BIO-RAD, Hercules, CA, USA). PCR was performed over 24 cycles of denaturation at 95 °C for 30 s, annealing at 60 °C for 30 s, and elongation at 72 °C for 30 s. The primer sequences used are shown in Table 1.

### 4.6. Reactive Oxygen Species (ROS) Measurement

RAW264.7 macrophages were seeded in 6-well plates at a density of 2 × 10^5^ cells per well and cultured overnight. After treated with the test compound for 1 h, LPS solution was added into the dish at a final concentration of 1 μg/mL and continually cultured for 24 h. The medium was removed and washed with PBS; DCFH-DA diluted in FBS-free medium to a final concentration of 100 μM was added and cultured at 37 °C for 30 min. The medium was removed, and the cells were washed once with PBS. The ROS expression lever was analyzed by using flow cytometry (Cytek, Aurora, CO, USA) and a fluorescence microscopy (Olympus, FV3000, Tokyo, Japan), with an excitation wavelength of 485 nm and an emission wavelength of 520 nm, respectively.

### 4.7. Tartrate-Resistant Acid Phosphatase (TRAP) Staining

RAW264.7 cells were seeded into 24-well plates at a density of 1 × 10^4^ cells per well and cultured overnight in α-MEM medium supplemented with 10% fetal bovine serum (FBS) and 100 mg/mL streptomycin, 2.5 mg/L amphotericin B. The cells were treated with different concentrations of Esa for 2 h and then added with 50 ng/mL recombinant murine soluble RANKL (Peprotech, Cranbury, NJ, USA). The cell suspension solution was replaced every two days. After 7 days, cells were fixed and stained using a TRAP activity staining kit (Wako, Osaka, Japan) according to the manufacturer’s protocol. Images after staining were acquired using a fluorescence microscope. TRAP-positive cells appeared dark red and TRAP-positive multinucleated cells with more than three nuclei were counted.

### 4.8. Immunofluorescence Staining

The cells were grown on an eight-chamber confocal dishes at a density of 5 × 10^3^ per well and treated with the test compounds for 24 h. Subsequently, the cells were fixed in 10% formalin solution for 15 min, washed three times with PBS, treated with 0.5% (*v*/*v*) Triton X-100/PBS for 15 min, washed three times with PBS again, and then blocked at room temperature for 30 min in 10% FBS/PBS. The cells were then incubated with rabbit anti NF-κB-p65 antibody (Cell Signaling Technology, Danvers, MA, USA) at 4 °C overnight, washed three times with PBS, incubated for 30 min at room temperature with secondary antibody anti-rabbit Alexa 488 (Immunoway, Plano, TX, USA), washed three times with PBS again, and then incubated with DAPI (5 mg/mL) at room temperature for 20 min. The localization of NF-κB-p65 was observed by using a confocal microscope (Olympus, FV3000, Tokyo, Japan) with an excitation wavelength of 496 nm and an emission wavelength of 519 nm.

### 4.9. Western Blot Analysis

Cells were harvested and suspended in lysis buffer, containing protease and phosphatase inhibitor cocktails. Nuclear protein extraction was performed using NE-PER^®^ nuclear and cytoplasmic extraction reagents (Thermo Scientific, Rockford, IL, USA). Concentrations of proteins were determined using a BCA protein assay (Thermo Scientific, Rockford, IL, USA). Equal amounts of proteins were resolved by 8% SDS-polyacrylamide gel electrophoresis and electrophoretically transferred to polyvinylidene difluoride (PVDF) membranes, which were then blocked in Tris-buffered saline containing 0.1% Tween 20 (TBS-T) and 5% skimmed milk for 1 h at room temperature, and then incubated with specific primary antibodies overnight at 4 °C.

### 4.10. Enzyme-Linked Immunosorbent Assay (ELISA) Analysis

The cell supernatant was collected and centrifuged at 4000 rpm to remove the sediment. Subsequently, the production of the inflammation-related indicators interleukin-6 (IL-6), interleukin-1 (IL1β), and tumor necrosis factor (TNF-α) was detected according to the instruction steps of the ELISA kit (Multisciences Biotech, Co., Ltd.,Hangzhou, China), and the absorbance value at 450 nm was measured by a microplate reader (PE, envision, US). Finally, the expression was calculated from the standard curve.

### 4.11. Statistical Analysis

The experiments were performed in triplicate and expressed as mean ± standard deviation (SD). The data were analyzed by one-way ANOVA. Data were considered statistically significant at *p*-values of <0.05.

## 5. Conclusions

**Figure 8:**
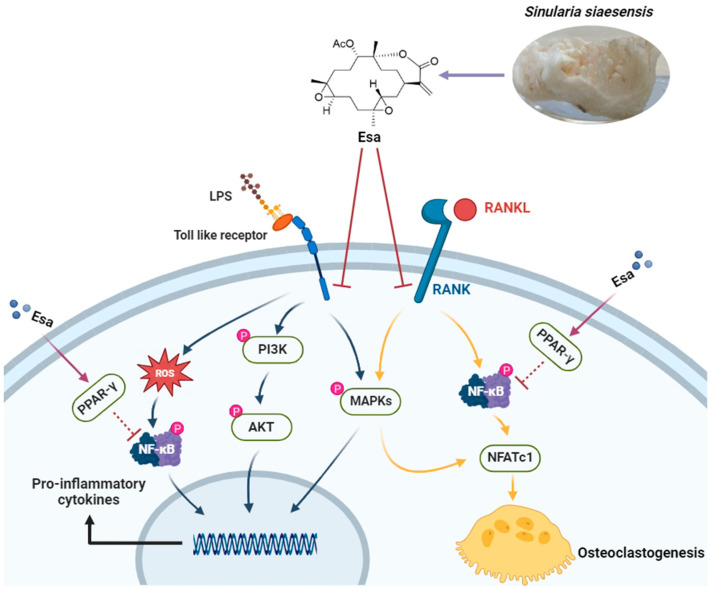
Diagram of hypothesized molecular effects of Esa contributing to inflammation and osteoclastogenesis.

In conclusion, this study demonstrates that the marine natural product Esa attenuates inflammation and osteoclastogenesis in vitro. Notably, Esa significantly inhibited LPS-induced inflammation via the suppression of pro-inflammatory protein release. Moreover, it inhibited RANKL-stimulated osteoclastogenesis by down-regulation of NF-κB and MAPK signaling pathways. It is worth noting that Esa promotes PPAR-γ expression in cell nuclei, which is possibly related to NF-κB suppression, as summarized in Figure 8. These results collectively exhibited that Esa could be used as a lead compound in drug development to treat osteoporosis.

## Figures and Tables

**Table 1:** List of primer sequences for quantitative PCR.

Prime Code	Prime Sequence (5’–3)
IL-6 forward	CTCCCAACAGACCTGTCTATAC
IL-6 reverse	CCATTGCACAACTCTTTTCTCA
IL-1β forward	CACTACAGGCTCCGAGATGAACAAC
IL-1β reverse	TGTCGTTGCTTGGTTCTCCTTGTAC
GAPDH forward	CAGGAGGCATTGCTGATGAT
GAPDH reverse	GAAGGCTGGGGCTCATTT

## Data Availability

The data presented in this study are available upon request from the corresponding author.

## Supplementary Materials

The following supporting information can be downloaded at: https://www.mdpi.com/article/10.3390/md22020095/s1, Figure S1: ^1^H NMR spectrum (400 MHz) of 7,8-epoxy-11-sinulariolide acetate in CDCl_3_. Figure S2: ^13^C NMR (125 MHz) of 7,8-epoxy-11-sinulariolide acetate in CDCl_3_. Figure S3: the mass spectrometry of 7,8-epoxy-11-sinulariolide acetate. Figures S4–S11: original Western blot images.
