# Supplementary material for: In Vitro Insights into the Role of 7,8-Epoxy-11-Sinulariolide Acetate Isolated from Soft Coral Sinularia siaesensis in the Potential Attenuation of Inflammation and Osteoclastogenesis
Source: Mar Drugs. 2024 Feb 19;22(2):95. doi: 10.3390/md22020095 (PMC10890379; doi:10.3390/md22020095)

Figure S1. ^{1}H NMR spectrum (400 MHz) of 7,8-epoxy-11-sinulariolide acetate in $CDCl_3$.

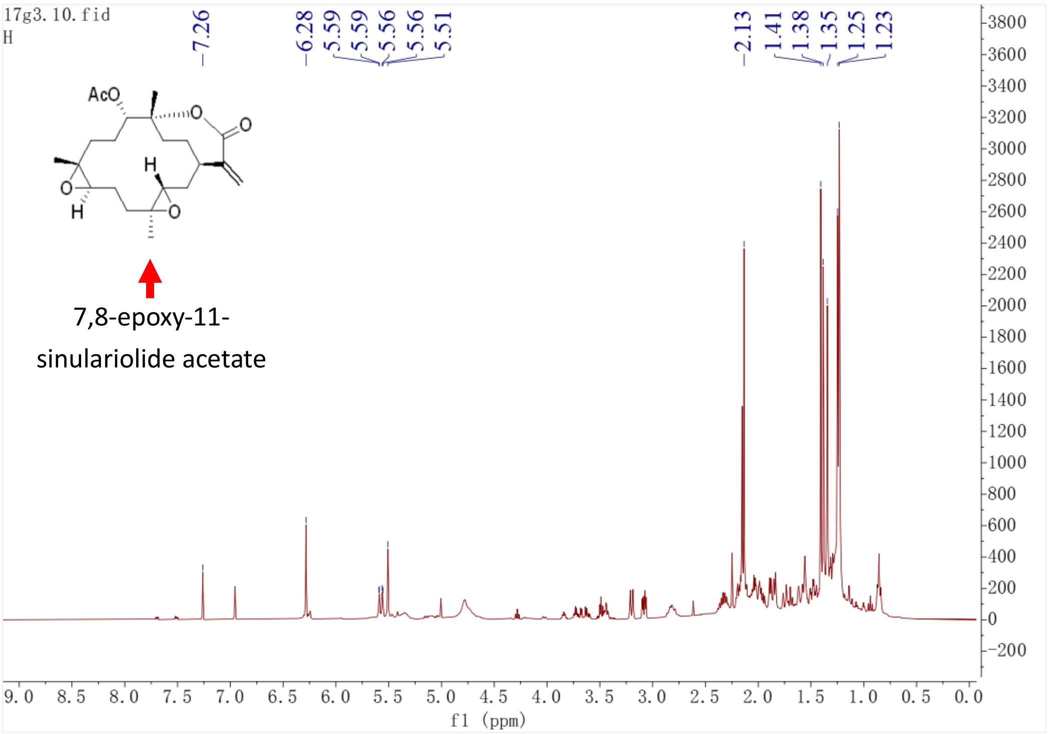

Figure S2. ^{13}C NMR (125 MHz) spectrum of 7,8-epoxy-11-sinulariolide acetate in $CDCl_3$.

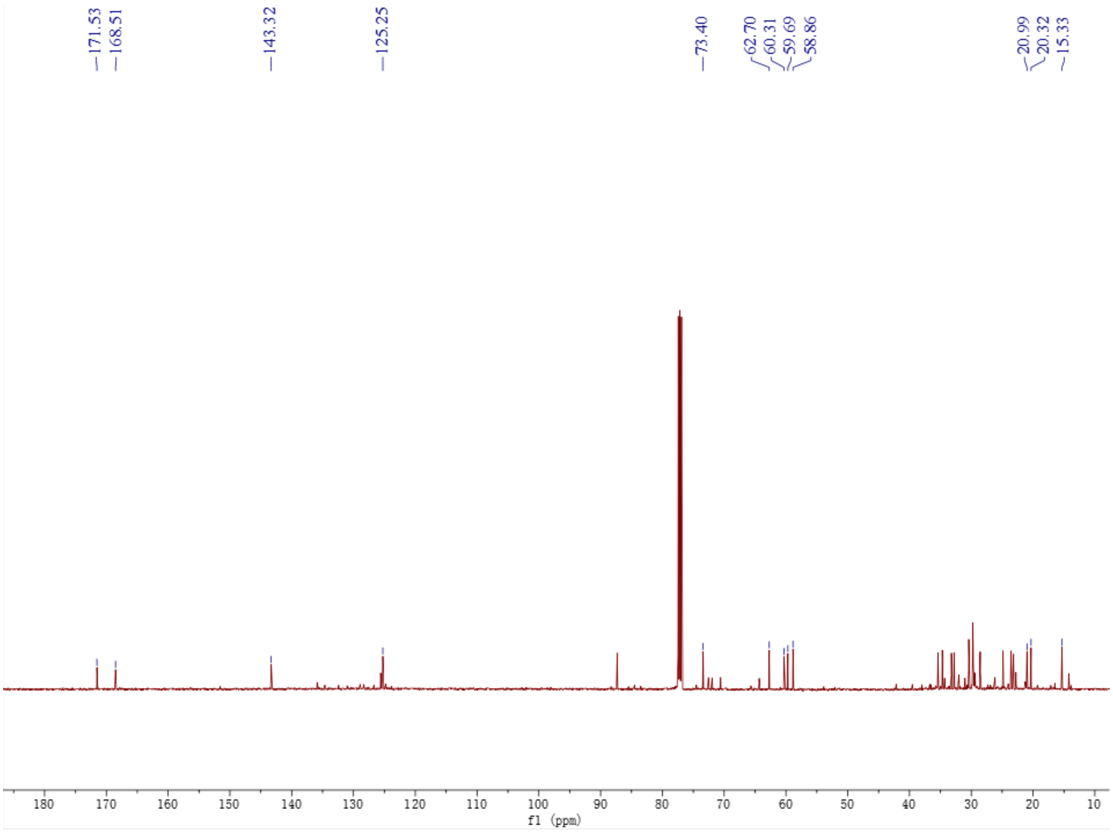

Figure S3. The Mass Spectrometry of 7,8-epoxy-11-sinulariolide acetate

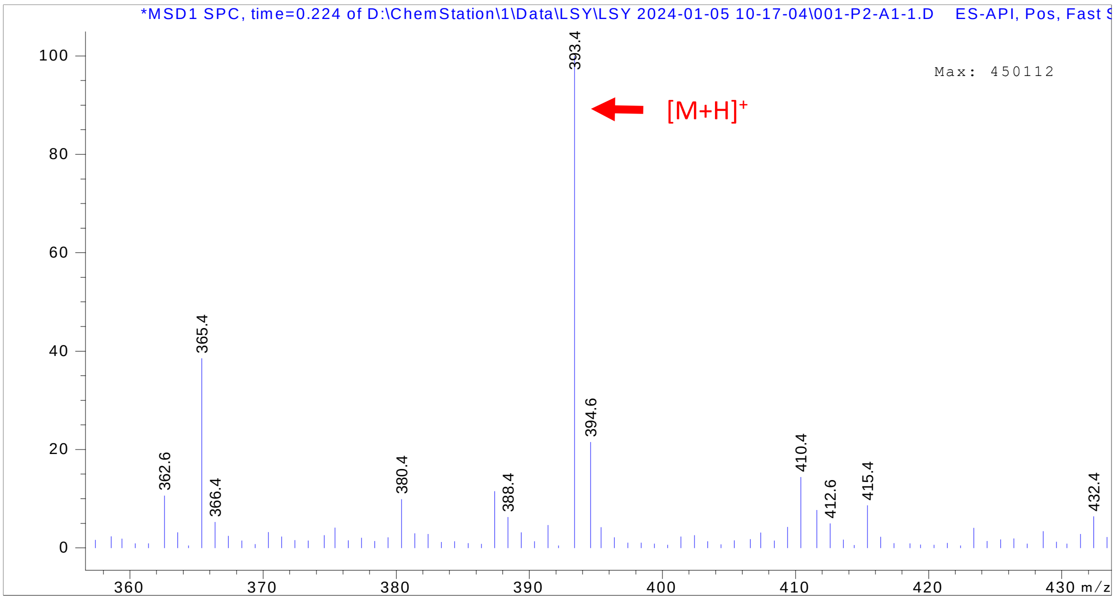

Figure S4. Original western blot images for three repeats of iNOS, COX-2, TNF-α and β-actin in Figure 2. (The six groups are CON, LPS, LPS with three concentrations ESA, and LPS with DEX)

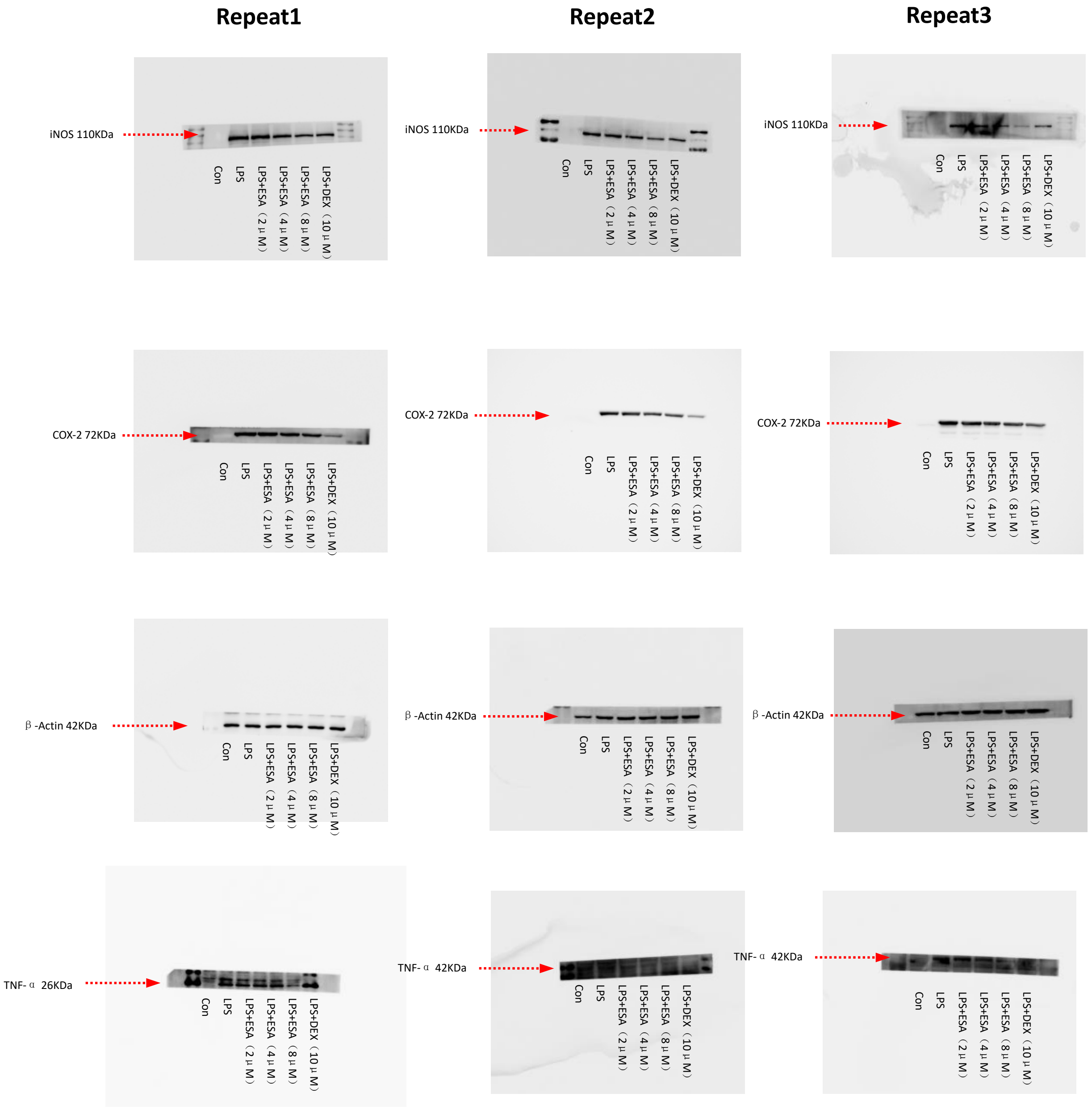

Figure S5. Original western blot images for three repeats of p-p65, p65 and GAPDH in Figure 4C. (The six groups are CON, LPS, LPS with three concentrations ESA, LPS with DEX)

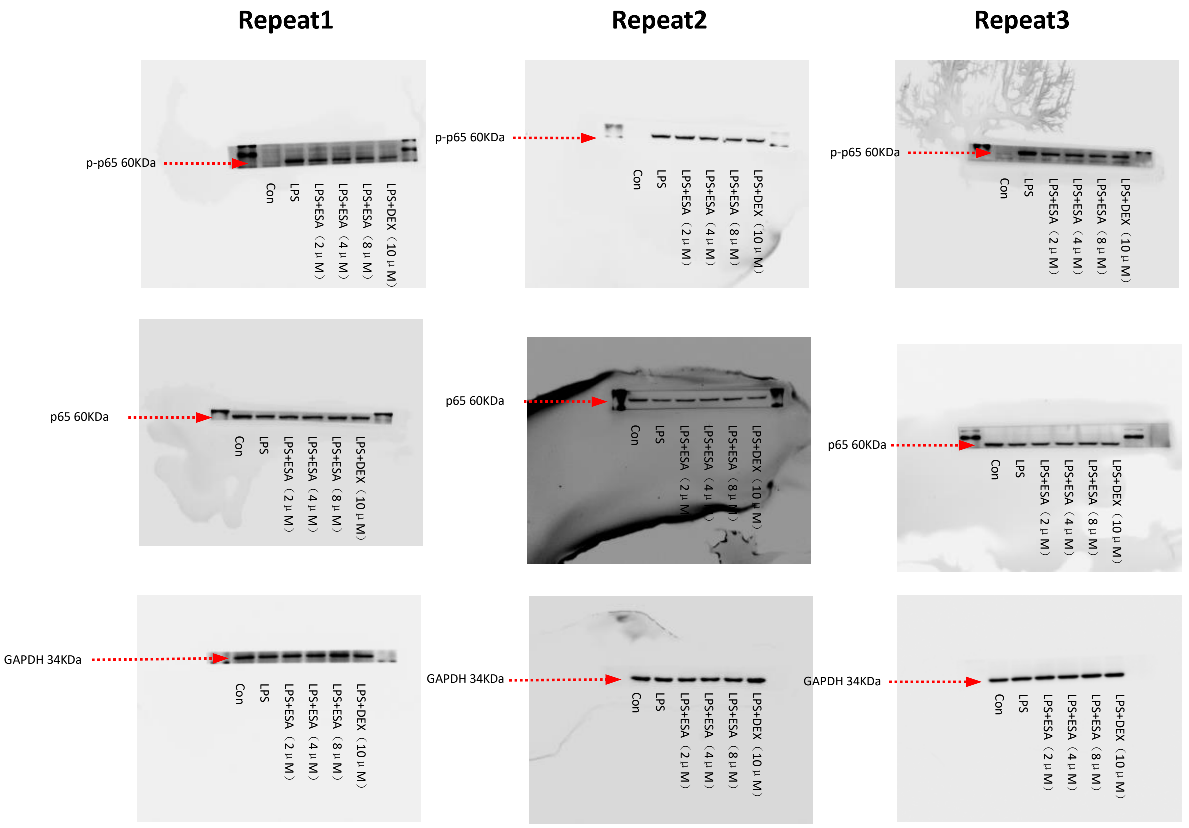

Figure S6. Original western blot images for three repeats of p-PI3K, p-JNK, p-AKT, p-p38 and GAPDH in Figure 5A-B. (The six groups are CON, LPS, LPS with three concentrations ESA, and LPS with DEX)

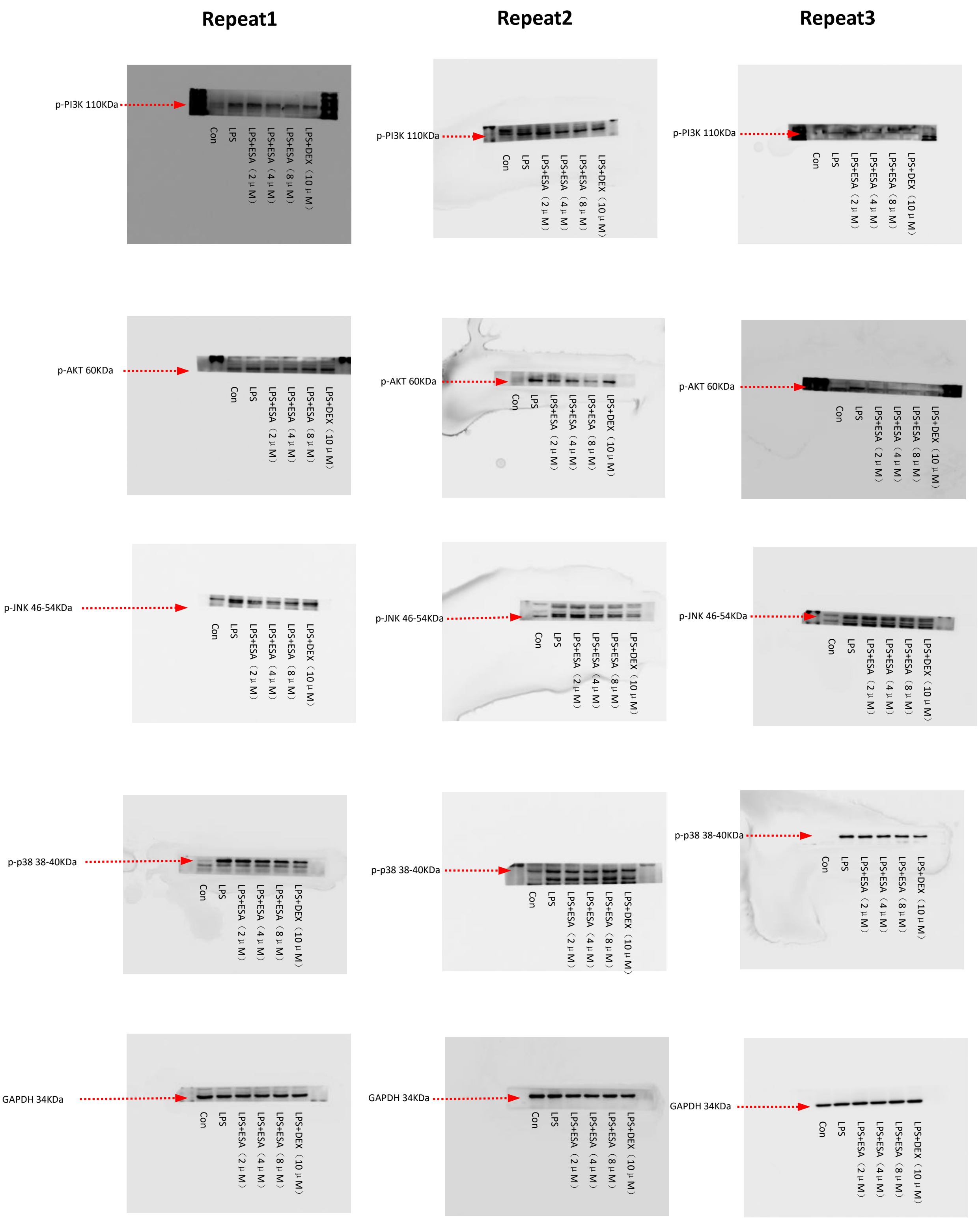

Figure S7. Original western blot images for three repeats of PI3K, AKT, JNK, P38 and GAPDH in Figure 5A-B. (The six groups are CON, LPS, LPS with three concentrations ESA, LPS with DEX)

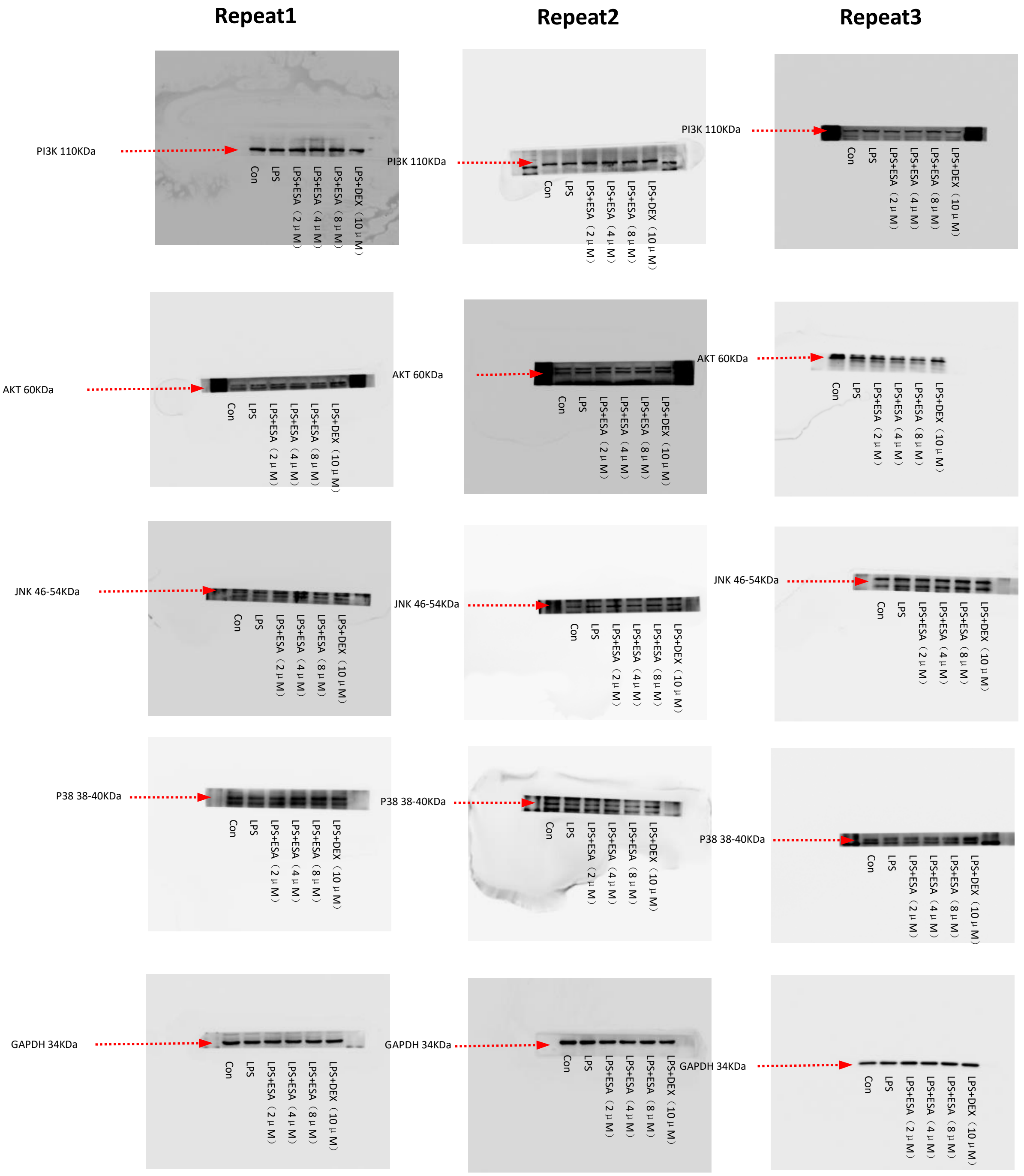

Figure S8. Original western blot images for three repeats of NFATc1, MMP9, CTSK and β-actin in Figure 6C. (The five groups are CON, RANKL, RANKL with three concentrations of ESA)

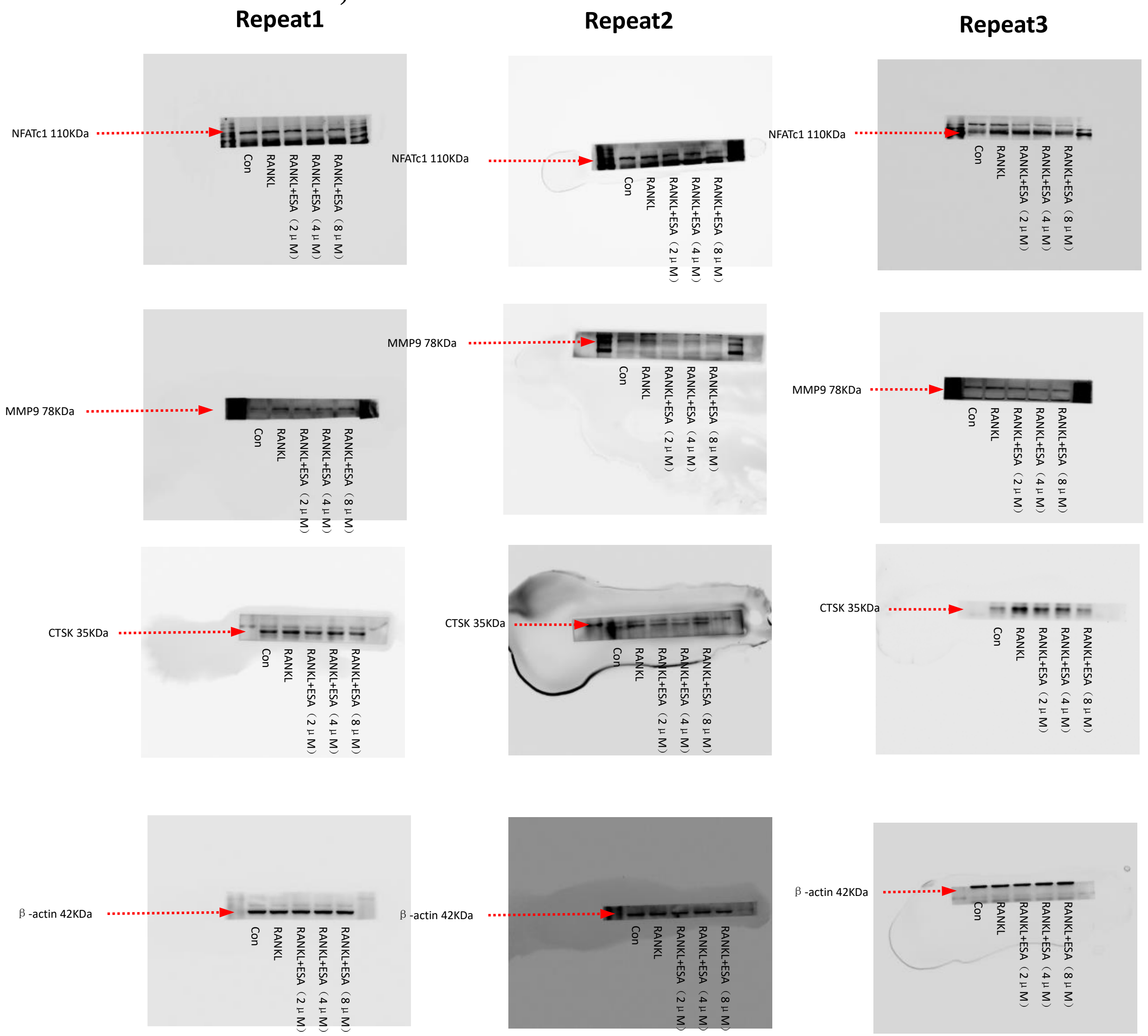

Figure S9. Original western blot images for three repeats of p-p65, p-JNK, p-p38, p-ERK in Figure 7A-B (The five groups are CON, RANKL, RANKL with three concentrations of ESA)

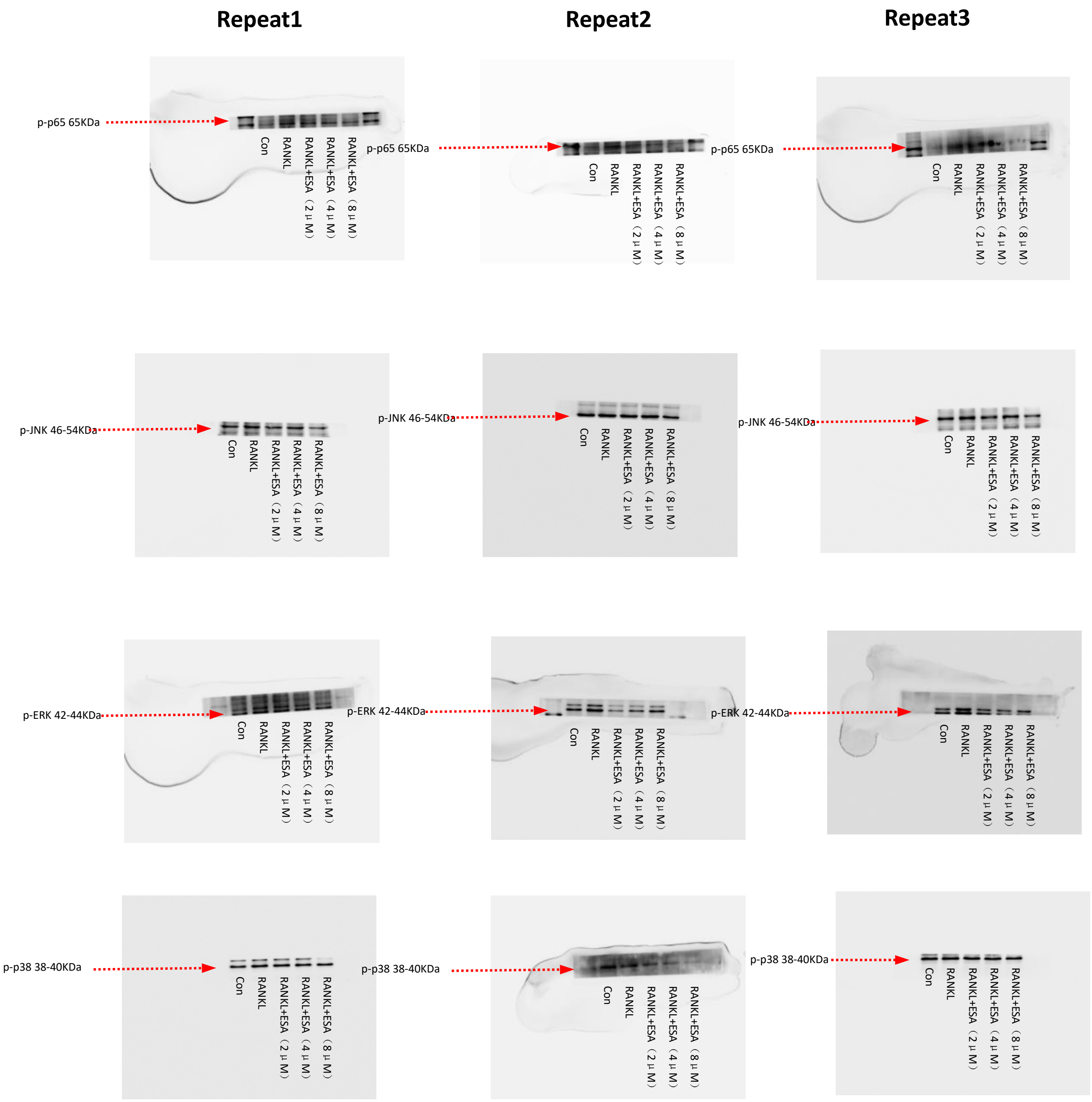

Figure S10. Original western blot images for three repeats of p65, JNK, p38, ERK and GAPDH/α-tubulin in Figure 7A-B. (The five groups are CON, RANKL, RANKL with three concentrations of ESA)

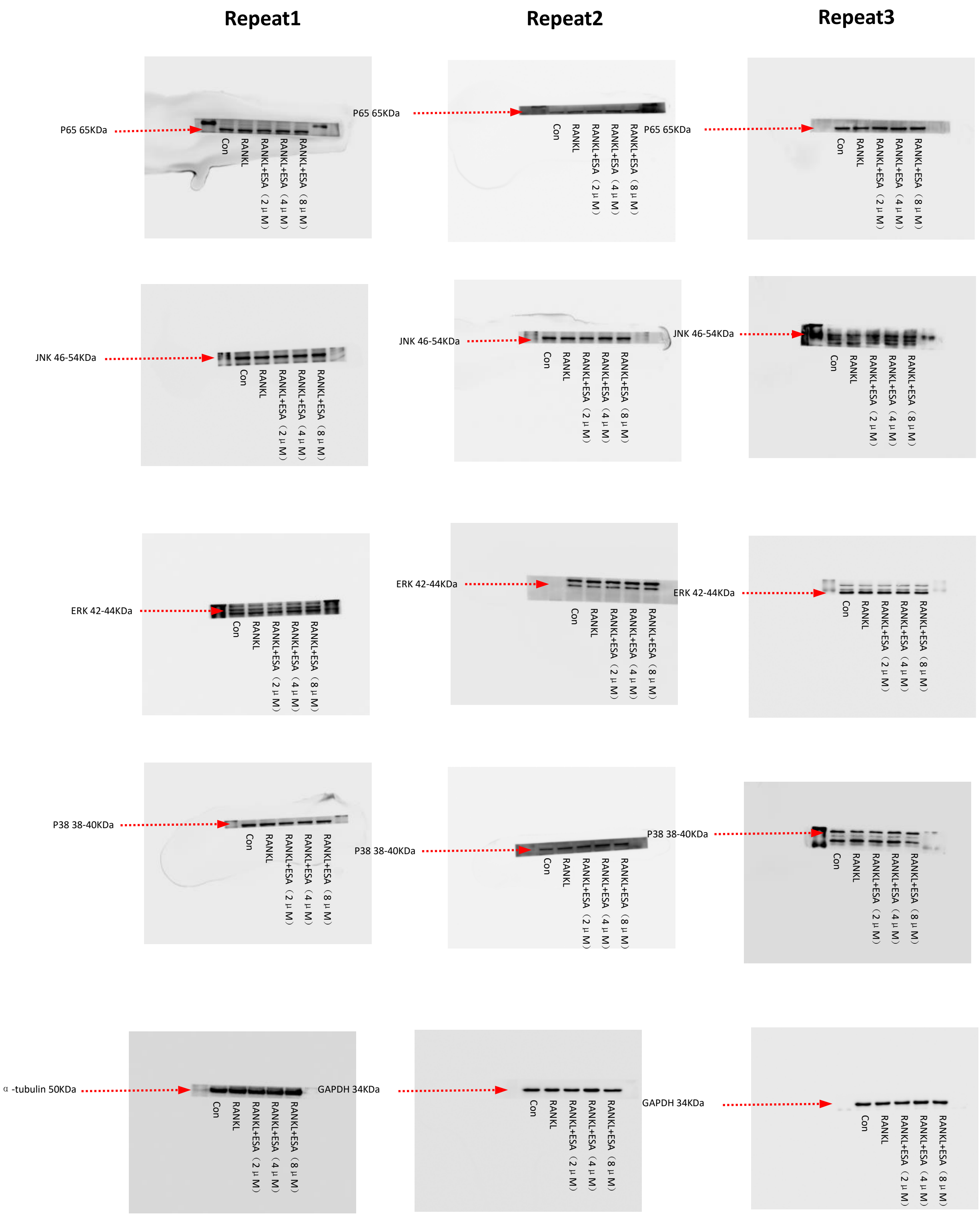

Figure S11. Original western blot images for three repeats of PPAR-γand Histone3 in Figure 7C. (The four groups are CON and three concentrations of ESA)

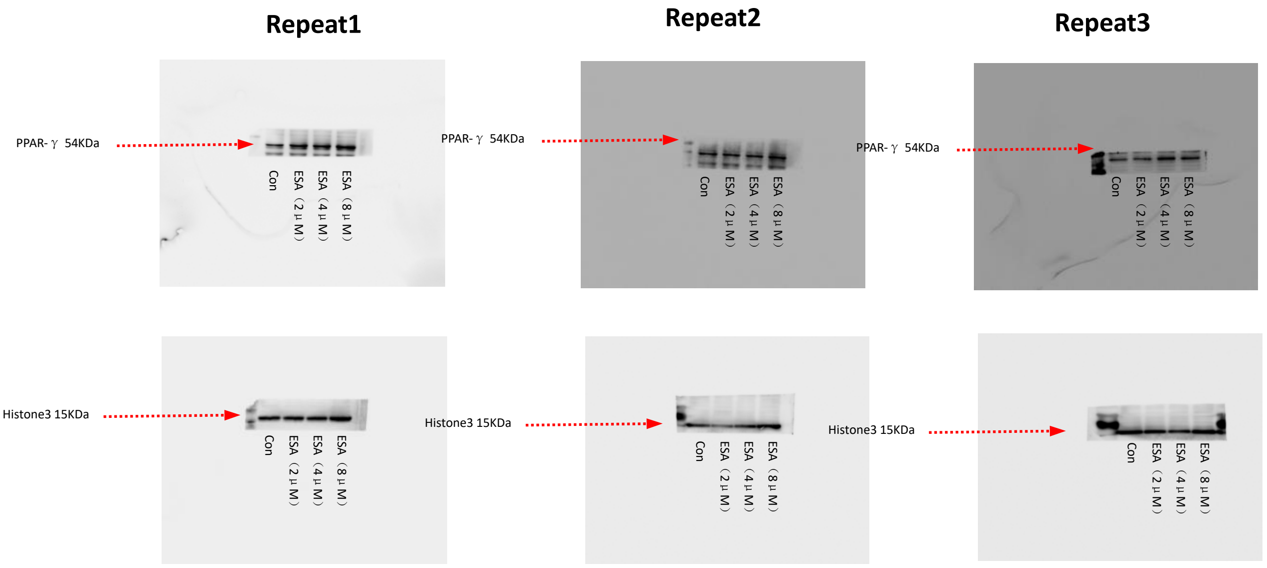

Supplement: Supplementary file 1 [file marinedrugs-22-00095-s001.zip › marinedrugs-2831850-supplementary.pdf]
